# Performance evaluation of protocols for *Taenia saginata* and *Ascaris suum* egg recovery from the house fly’s gastrointestinal tract and exoskeleton

**DOI:** 10.1186/s13071-023-06077-5

**Published:** 2023-12-18

**Authors:** Sophie De Bock, Luc Duchateau, Bruno Levecke, Sarah Gabriël

**Affiliations:** 1 Department of Translational Physiology, Infectiology and Public Health, Faculty of Veterinary Medicine, Ghent University, Salisburylaan 133, 9820 Merelbeke, Belgium; 2 Biometrics Research Group, Faculty of Veterinary Medicine, Ghent University, Salisburylaan 133, 9820 Merelbeke, Belgium

**Keywords:** *Taenia*, *Ascaris*, Helminth eggs, Environmental contamination, House flies, Dispersal, Recovery protocol, Recovery rate

## Abstract

**Background:**

The synanthropic house fly (*Musca domestica*) can potentially contribute to the mechanical spread of eggs of *Taenia* and *Ascaris* spp. in the environment and between hosts. However, the absence of validated protocols to recover eggs hampers an in-depth analysis of the house fly's role in parasite egg transmission.

**Methods:**

The gastrointestinal tract and exoskeleton of euthanized house flies were spiked with *Taenia saginata* eggs. The performance of several recovery protocols, in terms of both the recovery rate and ease-of-use, was (microscopically) evaluated and compared. These protocols employed steps such as washing, maceration, filtration, flotation and both passive and centrifugal sedimentation. The final validated protocols were subsequently evaluated for the recovery of *Ascaris suum* eggs.

**Results:**

The final protocol validated for the recovery of *T. saginata* eggs from the house fly’s gastrointestinal tract involved homogenization in phosphate-buffered saline and centrifugation at 2000 *g* for 2 min, yielding a recovery rate of 79.7%. This protocol required 6.5 min to perform (which included 1.5 min of hands-on time) and removed large debris particles that could hinder the differentiation of eggs from debris. Similarly, the final protocol validated for the recovery of *T. saginata* eggs from the fly’s exoskeleton involved washing by vortexing for 2 min in Tween 80 (0.05%), 15 min of passive sedimentation and centrifugation at 2000 *g* for 2 min, yielding a recovery rate of 77.4%. This protocol required 20.5 min to perform (which included 3.5 min of hands-on time) and successfully removed debris. The same protocols yielded recovery rates of 74.2% and 91.5% for the recovery of *A. suum* eggs from the fly’s gastrointestinal tract and exoskeleton, respectively.

**Conclusions:**

Effective, simple and easy-to-use protocols were developed and validated for the recovery of *T. saginata* and *A. suum* eggs from the house fly’s gastrointestinal tract and exoskeleton. These protocols can be applied to investigate the importance of flies as parasite egg transmitters in laboratory and field settings.

**Graphical Abstract:**

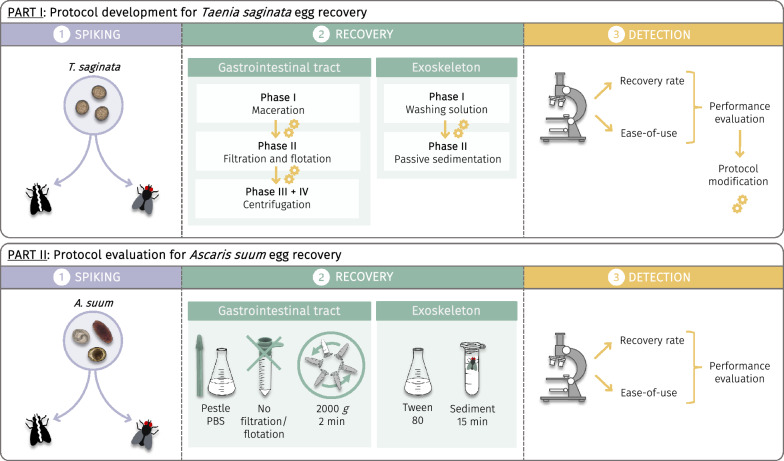

**Supplementary Information:**

The online version contains supplementary material available at 10.1186/s13071-023-06077-5.

## Background

Flies are mechanical carriers of pathogens, including fungi, bacteria, viruses and parasites. They facilitate the spread of pathogens into the environment, thereby increasing the risk of reaching susceptible hosts. Among all fly species, the house fly (*Musca domestica*) (Diptera: Muscidae) is the most abundant and cosmopolitan [1]. House flies can carry and transmit pathogens as they live in proximity to humans and animals, favor unsanitary environments and possess characteristic features, including sticky pads on their feet (pulvilli), hair-like structures on their bodies (setae) and a specific electrostatic charge. These characteristic features collectively promote the adhesion of pathogens on their exoskeleton [2, 3].

Fecal-borne helminths are particularly vulnerable to fly-mediated transmission, given the fly’s attraction to fecal matter for breeding and feeding purposes. Moreover, compared to other substrates, worm eggs embedded in highly viscous fecal matter are more likely to adhere to the exoskeleton of flies [2]. Through contact with infected stool or other contaminated surfaces, house flies can ingest parasite eggs and/or have them adhere to their exoskeleton. Consequently, eggs can directly reach susceptible hosts through accidental ingestion of the contaminated fly or indirectly through transportation to other substrates, such as food, water, soil and objects [4]. The eggs can be transmitted onto these substrates through physical dislodgment from the fly’s exoskeleton, regurgitation or fecal droppings [2].

Among helminths with a fecal–oral life cycle are *Taenia* and *Ascaris* spp., whose highly resistant eggs are abundantly excreted in the environment via stool [5, 6]. Ingestion of viable eggs from the zoonotic cestodes *Taenia saginata* and *Taenia solium*, excreted by a human tapeworm carrier, causes cysticercosis in cattle and pigs, respectively [7]. In contrast to *T. saginata*, *T. solium* can also cause cysticercosis in humans when its eggs are accidentally ingested. The establishment of *T. solium* cysts in the human central nervous system results in neurocysticercosis, which is a leading cause of preventable epilepsy [8]. Furthermore, the ingestion of embryonated eggs of *Ascaris lumbricoides*, and likely also *Ascaris suum*, which are soil-transmitted nematodes, causes ascariasis in humans [9]. Ascariasis mainly affects children, leading to intestinal obstruction, malnutrition, stunted growth and cognitive deficits [6]. *Taenia solium* cysticercosis and ascariasis are neglected tropical diseases which are endemic in areas characterized by unsanitary conditions and open defecation practices [10]. Unfortunately, the presence of these risk factors provides an ideal setting for the fly-mediated transmission of parasite eggs.

Considering the veterinary and public health implications associated with the ingestion of *T. solium* and *A. lumbricoides*/*suum* eggs by animals and humans, it is crucial to understand the mechanisms underlying the dispersal of eggs in the environment, including the possible role of flies and other insects. To date, inconsistent results have been reported regarding the carrier role of flies for *Taenia* and *Ascaris* spp. eggs [4, 11]. These studies applied various recovery techniques which involved steps to isolate and subsequently concentrate eggs from the fly matrix [12–25]. However, in fact, the application of diverse and unvalidated recovery techniques hampers the correct interpretation and comparison of interstudy results. Moreover, detailed descriptions of these techniques and the rationale underlying their implementation often remain undisclosed [26]. Also, several of these techniques can influence the viability of parasite eggs. Yet, viability assessment becomes crucial in determining the role of flies in parasite egg transmission, as the presence of eggs or egg DNA does not guarantee the presence of infective eggs.

Essentially, validated recovery protocols, which are currently lacking, should be applied to unravel the true role of flies in the mechanical transmission of parasite eggs. Ideally, these protocols are reliable and easy-to-use while still allowing the determination of egg viability. Hence, this study aimed to evaluate and validate protocols to recover and detect *T. saginata* eggs present in/on house flies. Specifically, the gastrointestinal tract and exoskeleton of house flies were spiked with eggs, after which the performance of different recovery protocols was compared. The performance evaluation, which considered both the recovery rate and ease-of-use of the protocol, served as a basis for further protocol modification. These protocols encompassed varying washing, maceration, filtration, flotation and both passive and centrifugal sedimentation steps. The final protocols were first validated for their effectiveness in recovering eggs from at least two different *T. saginata* egg batches. Thereafter, the protocols validated for recovering *T. saginata* eggs were evaluated for the recovery of *A. suum* eggs from the gastrointestinal tract and exoskeleton of house flies.

## Methods

### Source of house flies, *T. saginata* eggs and *A. suum* eggs

Adult house flies were obtained from the stock colony maintained at room temperature under a natural photoperiod in the Laboratory of Foodborne Parasitic Zoonoses, Faculty of Veterinary Medicine (Ghent University, Merelbeke, Belgium). Flies were euthanized by freezing at – 20 °C for 15 min.

**Fig. 1:**
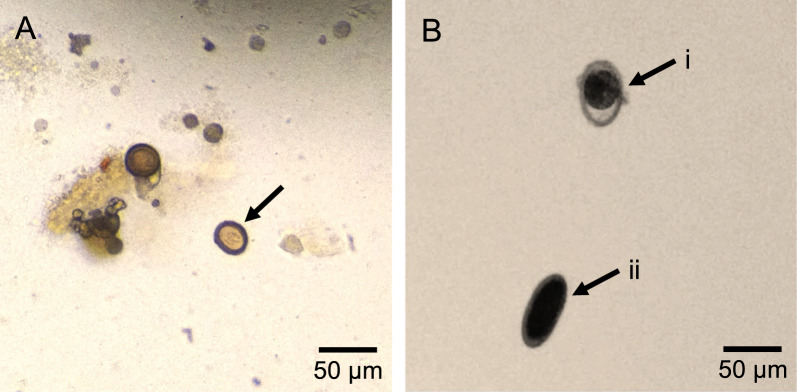
Intact eggs used for spiking (magnification ×200). **A** Intact *Taenia saginata* egg, **B** intact fertilized (i) and unfertilized (ii) *Ascaris suum* egg

*Taenia saginata* proglottids were obtained from Belgian commercial laboratories. Upon arrival, eggs were extracted from the proglottids by gentle maceration using forceps. The extracted eggs were stored in a solution containing phosphate-buffered saline (PBS) and antibiotics at 4 °C. During the experiment, multiple batches of *T. saginata* eggs were prepared. Each batch consisted of eggs from one worm due to the limited availability of fresh proglottids/worms. For each batch, the concentration and integrity of the eggs were microscopically evaluated. Eggs were considered intact if they were ovoid, measured 30–35 μm and contained an uninterrupted, radially striated embryophore. Only batches with intact eggs were used in the spiking experiment (Fig. 1).

*Ascaris suum* eggs were retrieved from the Laboratory of Parasitology, Faculty of Veterinary Medicine (Ghent University). The eggs had been isolated from multiple adult worms and stored in 2% potassium dichromate at room temperature. Prior to being used in the spiking experiment, the eggs were washed 3 times in PBS. The egg concentration and integrity were microscopically evaluated taking into consideration the visual characteristics described by Steinbaum et al. [27]. Intact fertilized and unfertilized eggs were used in the spiking experiment (Fig. 1).

### Protocols to recover *T. saginata* eggs from the gastrointestinal tract of house flies

The evaluation of recovery protocols comprised three major components: (i) spiking; (ii) recovery; and (iii) microscopic detection. First, the gastrointestinal tracts were microscopically removed using the dissection protocol described by Marchetti et al. [28] and transferred to 1.5-ml tubes, followed by gentle homogenization of the gastrointestinal tracts using a microtube pestle to release the gut contents. The gut homogenates were then spiked with three to nine *T. saginata* eggs. For this purpose, a 10-μl droplet of an egg batch with a mean concentration of 500 eggs/ml was placed on a microscope slide. After the eggs in the droplet were counted, they were again picked up using a micropipette and added to the gut homogenate. The remaining liquid on the microscope slide was then reassessed to confirm the number of eggs spiked. The spiked gut homogenates were stored at 4 °C.

**Fig. 2:**
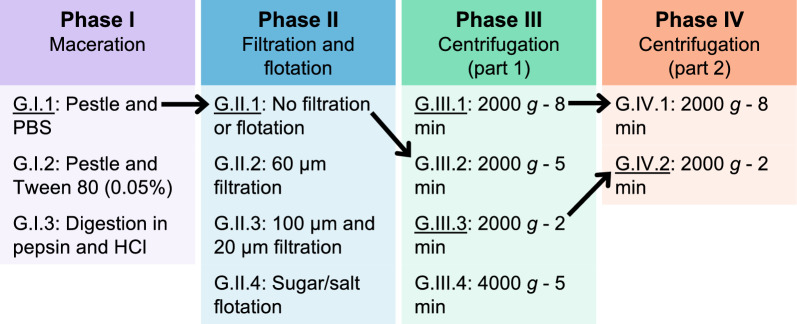
Four-phase systematic assessment and modification of protocols for the recovery of *Taenia saginata* eggs from the gastrointestinal tract of house flies. The black arrows indicate the best-performing protocols, as determined by performance evaluation, which were repeated in the subsequent phase. Protocol notation: G, gastrointestinal tract; I–IV, phase during which the protocol was evaluated; 1–4, protocol evaluated within that phase. PBS, Phosphate-buffered saline

One day after spiking, eggs were retrieved with different recovery protocols (Fig. 2). The protocol assessment comprised four phases (I–IV) in which various isolation and concentration techniques were compared: comparison of maceration techniques (phase I), filtration and flotation techniques (phase II) and centrifugation settings (phase III and IV). The protocols from phases I, II and III were performed on 15 spiked gut homogenates, and those from phase IV were performed on 20 spiked gut homogenates. For each phase, the best-performing protocol was determined and modified, with the aim to further enhance its performance. In each subsequent phase, the best-performing protocol from the previous phase was repeated along with the modified protocols (as indicated in Fig. 2 by the black arrows between consecutive phases).

**Fig. 3:**
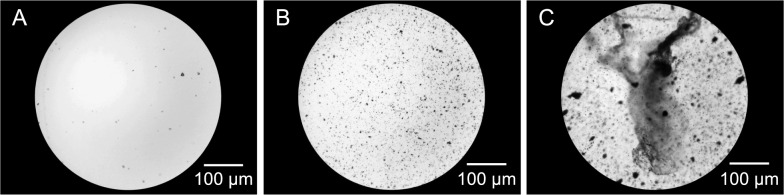
Slide readability based on scoring of microscopic debris (magnification ×100). **A** Slide with debris score of 1, indicating minimal debris. **B** Slide with debris score of 2, indicating some debris, necessitating additional time to enumerate the eggs. **C** Slide with debris score of 3, indicating too much debris, making it impossible to distinguish all eggs from debris

The performance of each protocol was evaluated based on the recovery rate and ease-of-use. To determine the recovery rate, eggs were enumerated under the microscope and the number compared to the initial number of eggs spiked. In all protocols except the flotation protocol, the supernatant was discarded following centrifugation, and the precipitate was then resuspended in the remaining solution and pipetted onto a clean microscope slide for microscopic enumeration. After performing the flotation protocol, the coverslip was placed on a clean microscope slide, and eggs were enumerated. The ease-of-use evaluation took into account the time required to complete the protocol, including hands-on and waiting time, as well as the readability of the microscope slides. Slide readability was determined by scoring the debris present during microscopic enumeration (Fig. 3), with a score of 1 indicating minimal debris, allowing easy differentiation of eggs from debris; a score of 2 indicating some debris, requiring additional time to scan the slide but still allowing the differentiation of eggs from debris; and a score of 3 indicating too much debris, making it impossible to distinguish all eggs from debris.

The protocols evaluated in each of the phases (I: maceration; II: filtration and flotation; III/IV: centrifugation) are discussed in more detail in the following sections. These protocols are labeled using code names formatted as G.I–IV.1–4, where “G” represents the gastrointestinal tract, “I–IV” indicates the phase during which the protocol was evaluated and “1–4” designates the protocol within that phase.

#### Phase I: maceration

**Fig. 4:**
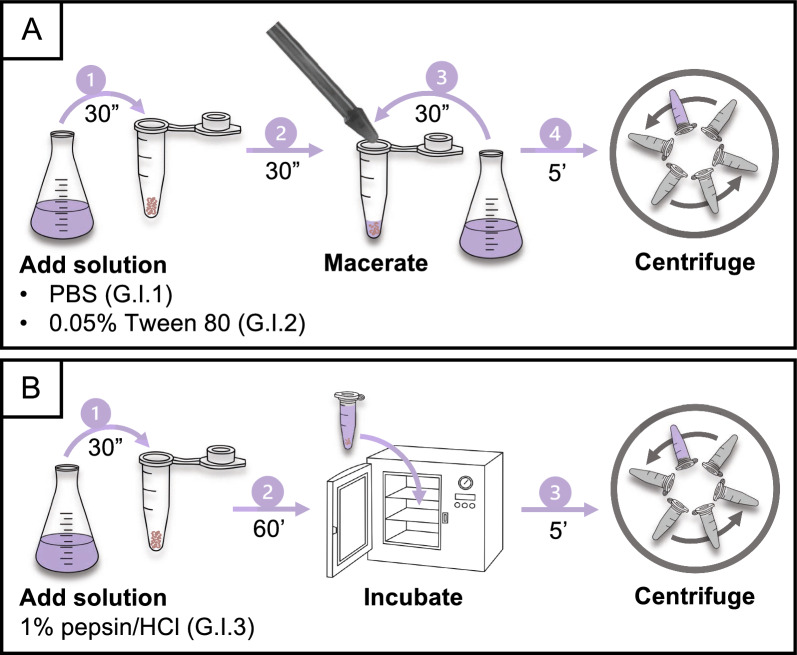
Protocols evaluated in the first phase (I: maceration) for the recovery of *Taenia saginata* eggs from the gastrointestinal tract, including the time required to perform each step. **A** The macerated gut homogenate was further homogenized using a pestle in PBS (G.I.1) or Tween 80 (G.I.2). The pestle was rinsed with the same solution utilized previously and the tube was centrifuged at 2000 *g* for 5 min. **B** The macerated gut homogenate was digested in 1% pepsin/HCl solution by incubation at 37 °C for 1 h (G.I.3). After incubation, the tube was centrifuged at 2000 *g* for 5 min. Protocol notation: G, gastrointestinal tract; I, phase during which the protocol was evaluated; 1–3, protocol evaluated within that phase. PBS*,* Phosphate-buffered saline

Three maceration protocols (G.I.1–3) were performed in the first phase (Fig. 4): two protocols assessed maceration with a pestle in different solutions, including PBS (G.I.1) and Tween 80 (0.05%) (G.I.2), and the third protocol assessed digestion of the gastrointestinal tract (G.I.3).

In the first two protocols (Fig. 4a), 100 μl of PBS (G.I.1) or 0.05% Tween 80 (G.I.2) was added to the tube containing the spiked gut homogenate (step 1). The gut homogenate was then macerated using a microtube pestle (step 2). To dislodge any eggs adhering to the pestle, the pestle was rinsed over the tube with 1 ml of PBS or Tween 80 (0.05%), respectively (step 3). Eventually, the tube was centrifuged at 2000 *g* for 5 min (step 4).

In the third protocol (G.I.3; Fig. 4b), 1.5 ml of the digestion solution containing pepsin and hydrochloric acid (1%) was added to the spiked gut homogenate (step 1). After mixing, the tube was incubated at 37 °C for 1 h (step 2). Following incubation, centrifugation was performed at 2000 *g* for 5 min (step 3).

#### Phase II: filtration and flotation

**Fig. 5:**
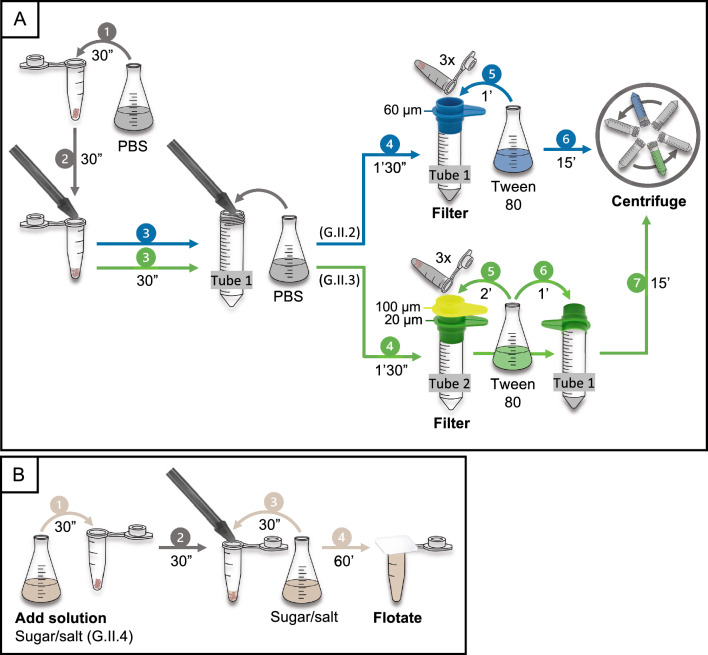
Protocols evaluated in the second phase (II: filtration and flotation) for the recovery of *Taenia saginata* eggs from the gastrointestinal tract, including the time required to perform each step. Gray arrows denote steps from the best-performing protocol of the previous phase, while the other colored arrows (blue, green or beige) denote modified steps. **A** A single-filtration protocol (G.II.2) and double-filtration protocol (G.II.3) were evaluated. In both protocols, filtration was performed after macerating the gut homogenate in PBS, through a 60-μm filter (for G.II.2) or through stacked 100-μm and 20-μm filters (for G.II.3), following which the filters were thoroughly rinsed with PBS/Tween 80. The 20-μm filter was rinsed an additional time after being inversely attached to a clean tube. Finally, centrifugation at 2000 *g* for 15 min was performed. **B** Protocol G.II.4 involved maceration of the gut homogenate in a sugar/salt solution, after which the gut homogenate was subjected to flotation for 1 h in the same solution. Protocol notation: G, gastrointestinal tract; II, phase during which the protocol was evaluated; 2–4, protocol evaluated within that phase. PBS, Phosphate-buffered saline

During the second phase, four protocols (G.II.1–4) were assessed: one without filtration or flotation step (G.II.1), two with a filtration step (G.II.2 and G.II.3) and one with a flotation step (G.II.4) (Fig. 5**)**.

Protocol G.II.1 was a repetition of protocol G.I.1 and remained unchanged during the second phase. Protocol G.II.2 and G.II.3 were repetitions of protocol G.I.1 that were extended with additional filtration steps (Fig. 5a). Briefly, after maceration in PBS (steps 1 and 2), the microtube pestle was rinsed with 1 ml of PBS over a clean 50-ml tube (step 3). In protocol G.II.2, the macerated gut homogenate and eggs in the 1.5-ml tube were filtered through a 60-μm filter that was connected to the same 50-ml tube used for rinsing the pestle (step 4: blue arrow). In protocol G.II.3, the contents of the 1.5-ml tube containing the macerated gut homogenate and eggs were filtered through a 100-μm and a 20-μm filter (step 4: green arrow). Both filters were connected to a 50-ml tube that was not the one used for rinsing the pestle. In both protocols, the 1.5-ml tube was refilled three consecutive times with 1 ml of PBS, briefly vortexed (3000 rpm for 5 s), and the solution passed through the filters to minimize egg loss in the tube. The filters were then rinsed with PBS/Tween 80 (0.05%), applying low pressure with a syringe that was attached to the filtration set-up to facilitate the straining of the sample (step 5). After rinsing both filters in protocol G.II.3, the upper 100-μm filter was removed and the 20-μm filter inversely attached to the 50-ml tube used for rinsing the pestle. Subsequently, the 20-μm filter was rinsed with PBS/Tween 80 (0.05%) (step 6: green arrow). Finally, the tube containing the recovered eggs was centrifuged at 2000 *g* for 15 min (step 6: blue arrow; step 7: green arrow).

Similar to protocols G.I.1/G.II.1, protocol G.II.4 also involved maceration with a pestle (Fig. 5b). However, instead of PBS, 100 μl of flotation solution containing sugar and salt (achieving a specific gravity of 1.30 at room temperature) was used for maceration (steps 1 and 2), followed by rinsing of the pestle in 1 ml of the same solution (step 3). Flotation solution was then added until a positive meniscus formed, which was topped with a coverslip (step 4). After 1 h, the coverslip was transferred to a clean microscope slide.

#### Phases III, IV: centrifugation

In phase III, the impact of two centrifugal forces and three centrifugation times on the performance was assessed, resulting in four protocols (G.III.1–4). Protocol G.III.2 was a repetition of protocol G.II.1 and remained unchanged during the third phase. Furthermore, the same protocol was evaluated with three different centrifugation settings: 2000* g* for 8 min (G.III.1), 2000 *g* for 2 min (G.III.3) and 4000 *g* for 5 min (G.III.4). The two settings yielding the best performance, namely centrifugation at 2000 *g* for 8 min (G.IV.1) and at 2000 *g* for 2 min (G.IV.2), were then reassessed in the fourth phase (phase IV).

### Protocols to recover *T. saginata* eggs from the exoskeleton of house flies

**Fig. 6:**
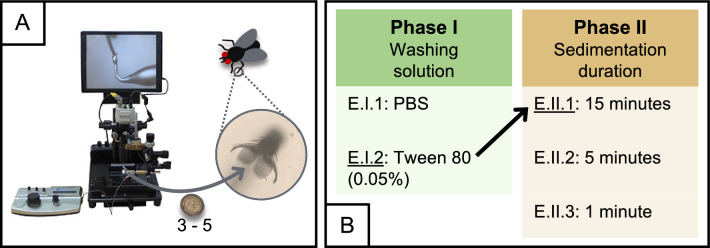
Procedure for the evaluation of protocols for the recovery of *Taenia saginata* eggs from the exoskeleton of house flies. **A** The pulvillus/claw region of each fly was spiked with 3 to 5 *T. saginata* eggs using the Micro Pick and Place System (Nepa Gene Co. Ltd.). **B** Two-phase systematic assessment and modification of the recovery protocols. The black arrow indicates the best-performing protocol, as determined by performance evaluation, which was repeated in the subsequent phase. Protocol notation: E, exoskeleton; I–II, phase during which the protocol was evaluated; 1–3, protocol within that phase. PBS, Phosphate-buffered saline

The evaluation of recovery protocols from the exoskeleton of house flies comprised three major components: (i) spiking; (ii) recovery; and (iii) microscopic detection. Initially, the pulvillus/claw region of each euthanized fly was spiked with approximately five *T. saginata* eggs using the Micro Pick and Place System (Nepa Gene Co. Ltd., Ichikawa, Chiba, Japan) (Fig. 6a). The pipette tip was examined carefully to verify that all eggs had been discharged. If fewer than three eggs were spiked, the fly was not included in the experiment. The spiked flies were stored at room temperature.

One day after spiking, eggs were retrieved using different recovery protocols. The assessment of the protocols comprised two phases: comparison of washing solutions (phase I) and passive sedimentation duration (phase II) (Fig. 6b). Each protocol was performed on 15 spiked flies. The protocols in phase II built upon the best-performing protocol from phase I, which is indicated by the black arrow between both phases. For each protocol, the microscopic recovery rate determination and ease-of-use evaluation was performed as described in section Protocols to recover* T. saginata* eggs from the gastrointestinal tract of house flies.

In the following sections, we discuss the protocols evaluated in each of the phases (I: washing solution; II: passive sedimentation duration) in more detail. The protocols are labeled using code names formatted as E.I–II.1–3, where “E” represents the exoskeleton, “I–II” indicates the phase during which the protocol was evaluated and “1–3” designates the protocol within that phase.

#### Phase I: washing solution

**Fig. 7:**
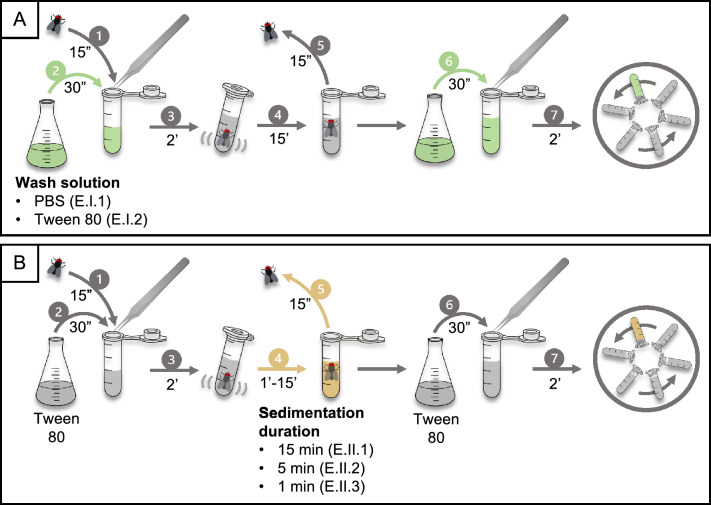
Protocols evaluated in both phases for the recovery of *Taenia saginata* eggs from the exoskeleton, including the time required to perform each step. **A** Comparison of two protocols involving different washing solutions, including PBS (E.I.1) and 0.05% Tween 80 (E.I.2). Following washing of the fly’s exoskeleton in either solution by vortexing, the fly was left undisturbed for 15 min before being removed from the tube. The contents of the tube were then centrifuged at 2000 *g* for 2 min. **B** The protocol was repeated as previously described (E.I.2); however, three different passive sedimentation durations were assessed: 15 (E.II.1), 5 (E.II.2) and 1 (E.II.3) min. Protocol notation: E, exoskeleton; I–II, phase during which the protocol was evaluated; 1–3, protocol within that phase. PBS, Phosphate-buffered saline

In the first phase, two washing solutions were compared for dislodging eggs from the exoskeleton: PBS (E.I.1) and 0.05% Tween 80 (E.I.2) (Fig. 7a). The protocols involved transferring the spiked fly using forceps to a 2-ml tube containing 1 ml of PBS or Tween 80 (0.05%) (step 1). The tip of the forceps was rinsed over the same tube with 0.5 ml of the respective washing solution (step 2). Next, the tube was vortexed at maximum speed (3000 rpm) for 2 min (step 3). The fly was then left undisturbed in the tube for 15 min to allow the eggs to settle (step 4). After passive sedimentation, the fly was removed from the tube using the same forceps (step 5), which was again rinsed over the same tube with 0.5 ml of PBS or Tween 80 (0.05%) (step 6). Finally, the homogenate, consisting of washing solution and any dislodged eggs, was centrifuged for 2 min at 2000 *g* (step 7).

#### Phase II: passive sedimentation duration

In phase II, three passive sedimentation durations of 15 (E.II.1), 5 (E.II.2) and 1 (E.II.3) min, respectively, were evaluated (Fig. 7b). Relatively short durations were assessed because flies float on the surface due to the surface tension of the solution and the exoskeleton has hydrophobic properties. The protocols were performed as described in section Phase I: washing solution, using Tween 80 (0.05%) as the washing solution while varying the durations of the passive sedimentation step.

### Protocols to recover *A. suum* eggs from the gastrointestinal tract and exoskeleton of house flies

The validated protocols for the recovery of *T. saginata* eggs from the gastrointestinal tract and exoskeleton of the house fly were evaluated for the recovery of *A. suum* eggs. Spiking, detection and evaluation of performance were conducted as described for the recovery of *T. saginata* eggs. In total, 17 gut homogenates and 17 fly pulvilli/claws were spiked with *A. suum* eggs, which were recovered 1 day after spiking.

### Data analysis

Statistical analysis was performed in SAS software (SAS Institute, Cary, NC, USA) using a generalized mixed model with binomially distributed error and logit link. The individual fly was introduced as random effect. Mean recovery rate estimates were calculated for each protocol, and the protocols were compared pairwise within each phase using a global significance level of 5% and adjusting the comparison-wise significance level by the Bonferroni method. All data is provided in Additional file 1: Dataset S1.

## Results

### Performance evaluation of the protocols to recover *T. saginata* eggs from the gastrointestinal tract of house flies

#### Phase I: maceration

**Fig. 8:**
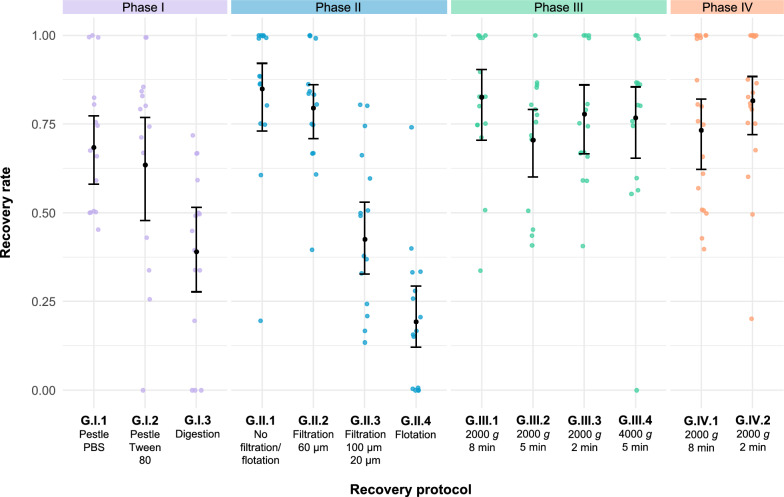
Individual and mean recovery rates yielded by the protocols for the recovery of *Taenia saginata* eggs from the gastrointestinal tract. Jittered points represent individual recovery rates. Mean recovery rates, estimated using a generalized mixed model with binomially distributed error and logit link, are plotted for each protocol. The error bars indicate the 95% confidence interval. Notation: G, gastrointestinal tract; I–IV, phase during which the protocol was evaluated; 1–4, protocol evaluated within that phase. PBS, Phosphate-buffered saline

During the first phase, two different egg batches were used for spiking. Recovery rates ranged from 39.0% (G.I.3) to 68.4% (G.I.1) (Table 1; Fig. 8). One sample subjected to maceration in Tween 80 was removed from the analysis due to spillage. The waiting times ranged from 5 min (G.I.1 and G.I.2) to 65 min (G.I.3), whereas the hands-on times ranged from 30 s (G.I.3) to 1 min and 30 s (G.I.1 and G.I.2). Debris scores of 2 (G.I.1 and G.I.2) and 3 (G.I.3) were assigned.

**Table 1:** Recovery rate and time to recover *Taenia saginata* eggs from the gastrointestinal tract of house flies for 13 protocols across four phases

Recovery protocol^a^	Sample size (*n*)	Spiking range	Eggs spiked, average	Flies with 100% recovery	Flies with 0% recovery	Recovery rate (95% CI)^b^	Time to recovery		Debris score^c^
							Waiting time	Hands-on time	
Phase I: maceration									
G.I.1	15	3–9	5.1	3	0	0.684a (0.580–0.773)	5’	1′30”	2
G.I.2	14	3–9	5.3	2	1	0.635a (0.478–0.768)	5’	1′30”	2
G.I.3	15	3–9	5.1	0	3	0.390b (0.277–0.515)	65’	30”	3
Phase II: filtration and flotation									
G.II.1	15	4–8	5.7	6	0	0.849a (0.730–0.921)	5’	1′30”	2
G.II.2	15	3–8	5.2	4	0	0.795a (0.708–0.861)	15’	4′00”	1
G.II.3	15	3–9	5.8	0	0	0.425b (0.327–0.530)	15’	6′00”	1
G.II.4	15	3–9	5.9	0	5	0.193c (0.121–0.293)	60’	1′30”	3
Phase III: centrifugation (part 1)									
G.III.1	15	4–9	6.1	5	0	0.826a (0.705–0.904)	8’	1′30”	2
G.III.2	14	4–9	6.3	1	0	0.705a (0.600–0.791)	5’	1′30”	2
G.III.3	15	3–9	5.4	4	0	0.778a (0.666–0.860)	2’	1′30”	2
G.III.4	15	4–9	6.3	3	1	0.768a (0.653–0.854)	5’	1′30”	2
Phase IV: centrifugation (part 2)									
G.IV.1	20	3–8	5.1	7	0	0.733a (0.622–0.820)	8’	1′30”	2
G.IV.2	20	3–8	5.2	7	0	0.816a (0.720–0.884)	2’	1′30”	2

′ Minutes, ″ seconds, *CI* confidence interval

^a^G, Gastrointestinal tract; I–IV, phase during which the protocol was evaluated; 1–4, protocol evaluated within that phase

^b^Recovery rates (ranging from 0 to 1) followed by different lowercase letters within each phase are significantly different from each other at* P* ≤ 0.05

^c^A debris score of 1 indicates minimal debris; 2, indicates some debris which necessitates longer reading time but still allows differentiating eggs from debris; 3, indicates a lot of debris which prevents differentiating eggs from debris

The protocols using a pestle in PBS (G.I.1) and Tween 80 (G.I.2) performed better compared to the digestion protocol (G.I.3) because they: (i) yielded significantly higher recovery rates; (ii) were considerably faster to perform; and (iii) generated improved debris scores. While no significant difference was observed between protocol G.I.1 and G.I.2, and the time to recovery was the same with both protocols, the former protocol offered slightly improved slide readability as the use of Tween 80 occasionally resulted in the formation of air bubbles. Given that protocol G.I.1 also yielded no flies with 0% egg recovery, it was considered the best-performing in this phase.

#### Phase II: filtration and flotation

During the second phase, two different egg batches were used for spiking. Recovery rates ranged from 19.3% (G.II.4) to 84.9% (G.II.1) (Table 1; Fig. 8). The waiting times ranged from 5 min (G.II.1) to 60 min (G.II.4), while the hands-on times ranged from 1 min and 30 s (G.II.1 and G.II.4) to 6 min (G.II.3). The debris scores ranged from 1 (G.II.2 and G.II.3) to 3 (G.II.4).

The recovery rate yielded by the protocol without filtration (G.II.1) differed significantly from those yielded by the double-filtration (G.II.3) and flotation protocol (G.II.4). Among the protocol without filtration (G.II.1) and the single-filtration protocol (G.II.2), the former protocol was faster to perform but rendered the sediment more difficult to examine. Nevertheless, the sediment generated by protocol G.II.2 was larger, requiring more time to microscopically examine. Additionally, while protocol G.II.1 and G.II.2 both yielded no flies with 0% egg recovery, protocol G.II.1 yielded the highest number of flies with 100% egg recovery. Thus, protocol G.II.1 was considered to be the best-performing protocol in this phase.

#### Phases III, IV: centrifugation

During the third phase, one egg batch was used for spiking. Similar recovery rates were yielded by the four evaluated protocols, which ranged from 70.5% (G.III.2) to 82.6% (G.III.1) (Table 1; Fig. 8). One sample subjected to centrifugation at 2000* g* for 5 min was removed from the analysis due to spillage. Whereas the waiting times differed according to the centrifugation times, ranging from 2 min (G.III.3) to 8 min (G.III.1), the hands-on time was 1 min and 30 s for all protocols. The debris was scored 2 for all protocols.

Since no significant difference in recovery rates was observed between the four protocols, the least time-consuming protocol (G.III.3) could be considered the best-performing in this phase. Both protocols G.III.1 and G.III.3 yielded no flies with 0% egg recovery and a relatively high number of flies with 100% egg recovery. To validate the comparable recovery rates and full recovery successes yielded by protocol G.III.1 and G.III.3, both protocols were repeated in the subsequent phase using a different egg batch.

In the fourth phase, centrifugation at 2000 *g* for 8 min (G.IV.1) and 2 min (G.IV.2) yielded recovery rates of 73.3% and 81.6%, respectively. Again, no significant difference was observed between the protocols; hence, the protocol involving centrifugation at 2000 *g* for 2 min (G.IV.2) was considered to be the final validated protocol.

### Performance evaluation of the protocols to recover *T. saginata* eggs from the exoskeleton of house flies

#### Phase I: washing solution

**Fig. 9:**
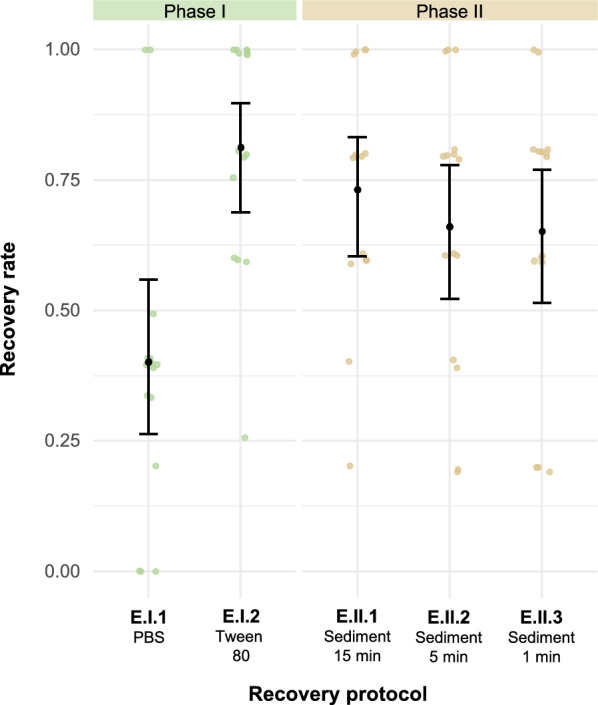
Individual and mean recovery rates yielded by the protocols for the recovery of *Taenia saginata* eggs from the exoskeleton. Jittered points represent individual recovery rates. Mean recovery rates, estimated using a generalized mixed model with binomially distributed error and logit link, are plotted for each protocol. The error bars indicate the 95% confidence interval. Protocol notation: E, exoskeleton; I–II, phase during which the protocol was evaluated; 1–3, protocol evaluated within that phase. PBS, Phosphate-buffered saline

During the first phase, one egg batch was used for spiking. The protocol using PBS (E.I.1) and Tween 80 (E.I.2) yielded significantly different recovery rates of 40.3% and 81.4%, respectively (Table 2; Fig. 9). For both protocols, waiting time was 17 min, hands-on time was 3 min and 30 s, and the debris was scored 1. Despite occasionally causing air bubble formation which slightly hindered the readability of the sediment, the protocol using Tween 80 (E.I.2) was considered to be the best-performing protocol in this phase.

**Table 2:** Recovery rate and time to recover *Taenia saginata* eggs from the exoskeleton of house flies for five protocols across two phases

Recovery protocol^a^	Sample size (*n*)	Spiking range	Eggs spiked, average	Flies with 100% recovery	Flies with 0% recovery	Recovery rate (95% CI)^b^	Time to recovery		Debris score^c^
							Waiting time	Hands-on time	
Phase I: washing solution									
E.I.1	15	3–5	4.5	2	3	0.403a (0.264–0.559)	17’	3′30”	1
E.I.2	15	4–5	4.7	7	0	0.814b (0.688–0.897)	17’	3′30”	1
Phase II: passive sedimentation duration									
E.II.1	15	5–5	5.0	4	0	0.733a (0.604–0.832)	17’	3′30”	1
E.II.2	15	4–5	5.9	3	0	0.662a (0.522–0.779)	7’	3′30”	1
E.II.3	15	5–5	5.0	2	0	0.653a (0.514–0.770)	3’	3′30”	1

*′* Minutes,* ″* seconds, *CI* confidence interval

^a^E, Exoskeleton; I–II, phase during which the protocol was evaluated; 1–3, protocol evaluated within that phase

^b^Recovery rates (ranging from 0 to 1) followed by different lowercase letters within each phase are significantly different from each other at* P* ≤ 0.05

^c^A debris score of 1 indicates minimal debris; 2, indicates some debris which necessitates longer reading time but still allows differentiating eggs from debris; 3, indicates a lot of debris which prevents differentiating eggs from debris

#### Phase II: passive sedimentation duration

During the second phase, one egg batch was used for spiking. The recovery rates ranged from 65.3% (E.II.3) to 73.3% (E.II.1) (Table 2**; **Fig. 9). Waiting times differed according to the passive sedimentation durations, ranging from 3 min (E.II.3) to 17 min (E.I.1), whereas the hands-on time was 3 min and 30 s for all protocols. The debris was scored 1 for all protocols.

Since no significant differences in recovery rates were observed among the three durations, the 1-min protocol could be considered the best-performing based on the ease-of-use evaluation. However, the 15-min protocol yielded a higher recovery rate and a greater number of flies with 100% egg recovery, possibly rendering this protocol a superior alternative. Altogether, considering the consistently high recovery rates yielded across both phases, during which two different egg batches were used, the 15-min protocol was considered the final validated protocol.

### Performance evaluation of the protocols to recover *A. suum* eggs from the gastrointestinal tract and exoskeleton of house flies

**Fig. 10:**
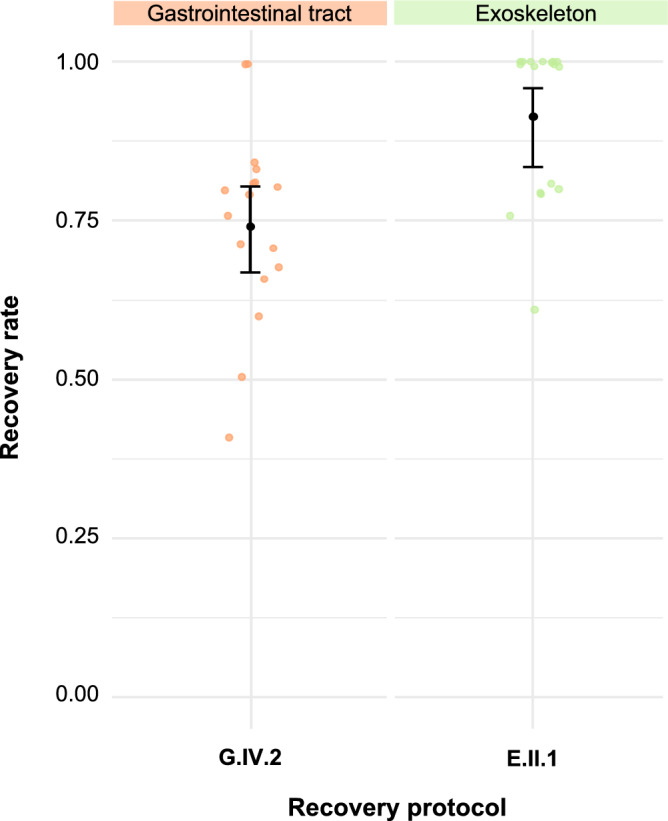
Individual and mean recovery rates yielded by the protocols for the recovery of *Ascaris suum* eggs from the gastrointestinal tract and exoskeleton. Jittered points represent individual recovery rates. Mean recovery rates, estimated using a generalized mixed model with binomially distributed error and logit link, are plotted for each protocol. The error bars indicate the 95% confidence interval. Protocol notation: E, exoskeleton; G, gastrointestinal tract; II, IV, phase during which the protocol was evaluated; 1,2, the protocol within that phase

To recover *A. suum* eggs from the gastrointestinal tract of house flies, the protocol that involved maceration with a pestle in PBS and centrifugation at 2000 *g* for 2 min (G.IV.2) was applied, yielding a recovery rate of 74.2% (Table 3; Fig. 10).

**Table 3:** Recovery rate and time to recover *Ascaris suum* eggs from the gastrointestinal tract and exoskeleton of house flies with the protocols validated for *Taenia saginata* egg recovery

Recovery protocol^a^	Sample size (*n*)	Spiking range	Eggs spiked, average	Flies with 100% recovery	Flies with 0% recovery	Recovery rate (95% CI)^b^	Time to recovery		Debris score^c^
							Waiting time	Hands-on time	
Gastrointestinal tract									
G.IV.2	17	3–7	5.2	2	0	0.742 (0.668–0.804)	2’	1′30”	2
Exoskeleton									
E.II.1	17	3–5	4.8	11	0	0.915 (0.834–0.958)	17’	3′30”	1

*′* Minutes,* ″* seconds, *CI* confidence interval

^a^G.IV.2: Gastrointestinal tract (G), phase IV, protocol 2. E.II.1: Exoskeleton (E), phase II, protocol 1

^b^Recovery rates range from 0 to 1.A debris score of 1 indicates minimal debris; 2, indicates some debris which necessitates longer reading time but still allows differentiating eggs from debris; 3, indicates a lot of debris which prevents differentiating eggs from debris

To recover *A. suum* eggs from the exoskeleton of house flies, the protocol that involved washing in Tween 80 (0.05%) and passive sedimentation for 15 min (E.II.1) was applied, yielding a recovery rate of 91.5% (Table 3; Fig. 10). The ease-of-use observations were consistent with those previously described for the recovery of *T. saginata* eggs.

## Discussion

In this study, validated laboratory protocols were established for the recovery of two types of helminth eggs from the gastrointestinal tract and exoskeleton of house flies. These protocols were selected for their superior performance results. Initially, a protocol was established for the recovery of *T. saginata* and *A. suum* eggs from the gastrointestinal tract, yielding recovery rates of 79.7% and 74.2%, respectively. This protocol involved homogenization with a pestle and centrifugal sedimentation. Among all of the protocols tested, the digestion and flotation protocol performed poorest in terms of recovery rate, time to recovery and debris accumulation. The digestion solution, intended to break down the gastrointestinal wall, has the potential to affect the embryophores of the eggs when it is taken into consideration that pepsin and hydrochloric acid have previously been used for hatching [29–31]. In addition, the debris formed during digestion largely hindered the identification of eggs. Furthermore, although a flotation solution with a specific gravity > 1.28 was used in which *Taenia* eggs normally float [32], eggs could have been resistant to floating due to the presence of debris or insufficient homogenization of the sample. Also, eggs adhering to other eggs or gut particles form heavier particles for which the flotation solution may become inadequate. Finally, crystallization made it a challenge to read the coverslips.

Surprisingly, while successful at removing debris from the final sediment, additional filtration steps failed to significantly improve egg recovery. Egg loss during filtration could have occurred due to the disruption of eggs by the pestle, clumping of eggs with other eggs or gut particles or difficulties in detaching eggs from the 20-μm filter. Specifically, disrupted eggs may pass through filters that would retain intact eggs (20-μm filter), while clumped eggs may be retained by filters through which individual eggs normally pass (60-μm and 100-μm filter). Efforts made to overcome these challenges, including gentle homogenization of the sediment and thorough rinsing of the filters, were not satisfactory.

One of the objectives of this study was to establish a balanced centrifugation setting that would prevent both the accumulation of debris from higher centrifugation force and/or time as well as continued suspension of eggs due to inadequate centrifugation force and/or time. We also aimed to identify time-saving centrifugation settings that would yield similar recovery rates. The final protocol involved centrifuging for 2 min, since increasing the centrifugation force to 4000 *g* or prolonging the centrifugation time to 8 min did not significantly improve egg recovery or alter the accumulation of debris. This finding is not surprising as it was previously reported that centrifuging at 1200 *g* for 3 min yields a satisfactory recovery of *Taenia* and *Ascaris* eggs in fecal samples [33].

While the final validated protocol yielded a satisfactory performance, it should be emphasized that the pestle disrupted some *T. saginata* eggs. Nevertheless, under natural conditions, eggs present inside the gastrointestinal tract would be partially protected from disruption. It is therefore essential to homogenize only for a short period of time, with the goal of releasing the gut contents, including eggs in naturally contaminated fly guts, without excessively disrupting the eggs.

The established protocol for the recovery of *T. saginata* and *A. suum* from the exoskeleton yielded recovery rates of 77.4% and 91.5%, respectively. This protocol included washing and (passive and centrifugal) sedimentation techniques. The two washing solutions were selected based on their widespread availability and low cost. Tween 80 was specifically selected for its detergent properties and frequent use in previous studies to recover eggs from various environmental matrices [26, 34]. We observed that washing with Tween 80 significantly improved the recovery of eggs compared to washing with PBS. It should be noted that while previous studies did not use detergents, they commonly used saline to wash *Taenia* spp., *Ascaris* spp. and other parasite eggs off the exoskeleton of flies [12–19], likely leading to underestimated egg counts.

A passive sedimentation step was included in the protocol because eggs become homogeneously distributed in the washing solution during vortexing and might therefore be lost with the immediate removal of the fly. No significant differences were observed between the three passive sedimentation durations, probably indicating rapid sedimentation of dislodged eggs following vortexing. The 15-min protocol was chosen as the final validated protocol due to its previously confirmed recovery rate. If the laboratory’s workflow would not allow this longer waiting time, the 1-min protocol may also be a well-performing alternative. Nonetheless, it is advised to first validate this faster protocol using a different batch of eggs.

*Ascaris suum* eggs were more easily recovered from the exoskeleton of house flies than *T. saginata* eggs, resulting in a remarkably high number of flies with 100% *A. suum* egg recovery. The underlying reason for these observations is unclear but could be due to chance, variations in batch integrity or morphological differences. For example, *Ascaris* eggs are larger (approximately 35 to 75 μm in length), have a lower specific gravity of 1.13 and exhibit different developmental stages, which can be corticated or decorticated [27]. It should be noted that the sticky nature of *Ascaris* [35] and *Taenia* [36] eggs probably represents a further challenge in their recovery, regardless of the applied recovery protocol. Accordingly, egg loss can occur by eggs sticking to forceps, tubes, pestles, filters and pipette tips. To minimize this risk, we recommend thoroughly rinsing all materials coming into contact with eggs.

Microscopy was employed to detect the recovered eggs, providing a low-cost and widely available tool that allows egg enumeration and viability assessment. However, accurate microscopic detection of eggs requires a clean sediment, necessitating the removal of debris during the recovery process. In contrast to the present study, previous studies have also focused on pooled samples [12–14, 17, 18], for which more complex recovery protocols may be required to remove debris, especially for gastrointestinal tracts. Intriguingly, although seldom used for pooled fly matrices [13, 18], filtration and flotation techniques are commonly used for recovering eggs from soil, water and food samples [26, 34]. Alternatively, to omit the need for debris removal, quantitative or digital PCR could be employed.

Overall, this study has three main limitations. The first is the variation in recovery rates that was observed across different time points. Notably, the same protocol was replicated in the first three phases of the experiment to recover *T. saginata* eggs from the gastrointestinal tract, yielding recovery rates of 68.4%, 84.9% and 70.5% in the first, second and third phase, respectively. This variation may have resulted from the differences in the egg batches used for spiking, as even eggs from the same batch can have varying viability levels at different time points [5]. Accordingly, fragile eggs, despite their intact appearance, would be more vulnerable to disruption and to being lost during the recovery process. The selection of visually intact eggs from homogenous egg batches ensured a reliable comparison of protocols. However, under field conditions, the viability levels and adhering properties of eggs might differ, resulting in varying absolute recovery rates. Another limitation to the present study is that eggs were spiked on the gut homogenate, which may not fully represent the natural condition where eggs are located inside the gastrointestinal tract. This aim of this approach was to simulate the natural condition, considering that eggs might adhere to gut particles, thereby influencing the recovery of eggs. The third limitation is that the reported recovery rates for the exoskeleton are based on eggs recovered from the pulvilli/claws of flies. Under natural conditions, eggs may be present in other regions for which the ease of recovery might differ. Nevertheless, the pulvilli/claws were targeted due to their likely contact with contaminated substrates and because they are a region from which it is challenging to recover eggs, considering that eggs could adhere to the sticky pulvilli. It should be noted that in the present study, flies were handled using forceps in a manner that specifically avoided spiked areas. However, in naturally contaminated flies, the position of eggs on the exoskeleton would be unknown, which emphasizes that forceps should be thoroughly rinsed.

Essentially, wider implementation of the established protocols can enhance the comparison between study results, bringing researchers closer to determining the actual role of flies in the mechanical spread of parasites. These protocols, based on *T. saginata* and *A. suum*, could also be used for recovering *T. solium* and *A. lumbricoides* eggs. However, distinguishing between *Taenia* spp. and *Ascaris* spp. is impossible by microscopy alone. While these protocols are probably also applicable to other parasites and fly species, we advise conducting an initial performance assessment.

## Conclusions

The recovery rate and ease-of-use of several protocols for the recovery of *T. saginata* and *A. suum* eggs from the house fly’s gastrointestinal tract and exoskeleton were evaluated. Based on these performance evaluations, the protocols were systematically modified for further improvement. Eventually, two validated protocols were selected for their superior performance, characterized by their simplicity, ease-of-use and satisfactory recovery results. These protocols can be applied to investigate the importance of flies as parasite egg dispersers in both laboratory and field settings.

### Supplementary Information

**Additional file 1: Dataset S1.** Recovery of *T. saginata* and *A. suum* eggs from the spiked gastrointestinal tract and exoskeleton of house flies using various recovery protocols.

## Data Availability

The datasets supporting the conclusions of this study are included within the article and its additional files (Additional file 1: Dataset S1).

## Abbreviation

PBS: Phosphate-buffered saline
